# Contents Changes of Triterpenic Acids, Nucleosides, Nucleobases, and Saccharides in Jujube (*Ziziphus jujuba*) Fruit During the Drying and Steaming Process

**DOI:** 10.3390/molecules201219852

**Published:** 2015-12-12

**Authors:** Sheng Guo, Jin-Ao Duan, Ying Zhang, Dawei Qian, Yuping Tang, Zhenhua Zhu, Hanqing Wang

**Affiliations:** 1 Jiangsu Collaborative Innovation Center of Chinese Medicinal Resources Industrialization, Nanjing University of Chinese Medicine, Nanjing 210023, China; guosheng@njucm.edu.cn (S.G.); nzyzyb@163.com (Y.Z.); qiandw@njutcm.edu.cn (D.Q.); yupingtang@njutcm.edu.cn (Y.T.); 04040416@163.com (Z.Z.); 2 College of Pharmacy, Ningxia Medical University, Yinchuan 750004, China; wwwhhq@163.com

**Keywords:** *Ziziphus jujuba*, triterpenic acid, nucleoside, saccharide, drying process, content variation of the active compounds

## Abstract

Chinese jujube (*Ziziphus jujuba*), a medicinal and edible plant, is widely consumed in Asian countries owing to the remarkable health activities of its fruits. To facilitate selection of the suitable processing method for jujube fruits, in this study their contents of triterpenic acids, nucleosides, nucleobases and saccharides after drying and steaming treatment were determined using ultra-high performance liquid chromatography and high performance liquid chromatography coupled with evaporative light scattering detector methods. The results showed that except for sucrose, the content levels of most analytes were increasing in the jujube fruits during drying treatment at 45 °C. The levels of cyclic nucleotides such as adenosine 3′,5′-cyclic monophosphate and guanosine 3′,5′-cyclic monophosphate, were significantly decreased after the fruits were steamed. Therefore, owing to the bioactivities of these components for human health, the dried fruits would be the better choice as medicinal material or functional food, and dried jujube fruit should not be further steamed.

## 1. Introduction

*Ziziphus jujuba* Mill. is indigenous to China and widely distributed in the temperate and subtropical areas of the northern hemisphere, especially the inland region of north China [1,2]. Its fruits are widely consumed in Asian countries as a food or food additive owing to their high nutritional value [3]. It has also been utilized as a traditional Chinese medicine for thousands of years due to its numerous bioactivities such as immunomodulating [4,5], gastrointestinal protective [6], hepatoprotective [7,8], anticancer [9,10], anti-oxidation [4,11], anti-inflammatory [12,13] and neuroprotective effects [14,15,16]. Previous phytochemical studies showed that jujube fruits are especially rich in triterpenic acids [17,18,19], nucleosides [20], flavonoids [21,22], phenolic acids [23,24], cerebrosides [25], sugars [26,27] and amino acids [28,29], which contribute significantly to their nutritional and functional values as well as taste. Recently, there is a considerable interest in these biologically active compounds due to their health benefit to alleviate chronic diseases [30,31].

To date, jujube fruits have mainly been consumed in many countries in their fresh state or after being dried. In addition, in the north of China they are also used after being steamed to improve their taste. There are reports that have investigated the changes of flavonoid, phenolic acid, organic acid and volatile compound content in jujube fruits after applying different drying methods in comparison with fresh ones [32,33]. The results showed that the above constituents varied widely after being dried with different methods, which could provide the data for the selection of the most suitable drying method for jujube fruits. However, besides these components, little information could be found about the effect of drying treatments on other types of compounds such as nucleosides, nucleobases and triterpenic acids contained in jujube fruits. In addition, the changes of the bioactive compounds in jujube fruits after being steamed were not investigated till now. Researches showed that triterpenic acids contained in jujube fruits exhibited multiple bioactivities including anticancer, hepatoprotective, anti-inflammatory and antimicrobial effects [19,34]. Nucleosides and nucleobases were reported to relate with various physiological processes in the body and exhibited antiplatelet aggregation, antiarrhythmic, antiseizure, antioxidant and antitumor activities, all of which are contributed to the human health [35]. Besides, sugars such as fructose, glucose and sucrose are the main nutrients of jujube fruit and also contribute to its taste [32]. Therefore, to investigate the content variation and the change trends of the above three types of compounds in jujube fruits during drying or steaming treatment, would be helpful to evaluate their nutritional and bioactive values, and provide beneficial information for consumers to select the most suitable product.

**Figure 1:**
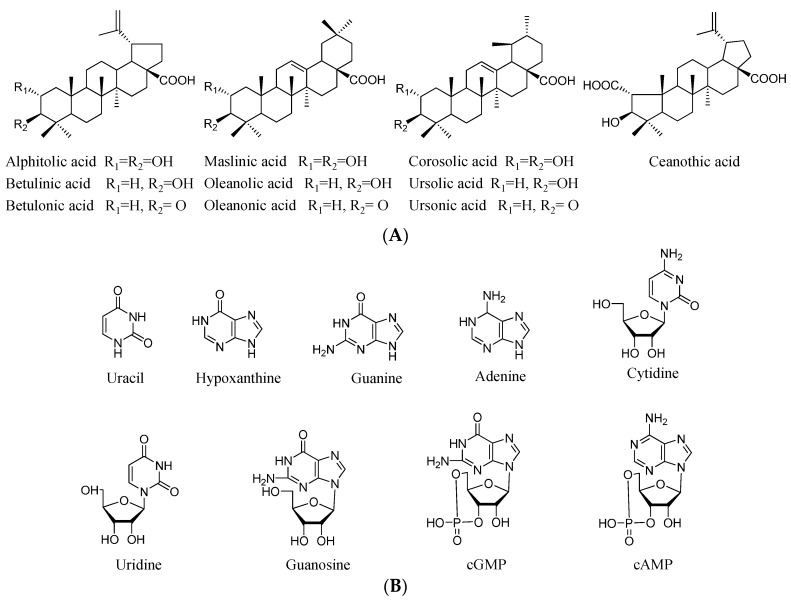

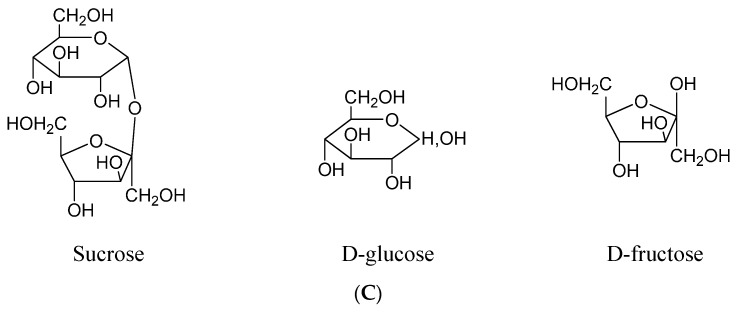
Structures of triterpenic acids, nucleosides and nucleobases as well as saccharides in jujube fruits evaluated in the present study. (**A**) Triterpenic acids; (**B**) Nucleosides and nucleobases; (**C**) Saccharides.

Therefore, to facilitate selection of a suitable jujube fruit processing method for providing optimum benefits as a health food and crude drug, in the study, triterpenic acids, nucleosides and nucleobases as well as saccharides (Figure 1) were determined in jujube fruits after drying for different times or after being steamed and their contents were compared using ultra-high performance liquid chromatography (UHPLC) and high performance liquid chromatography coupled with evaporative light scattering detector (HPLC-ELSD).

## 2. Results and Discussion

### 2.1. Triterpenic Acid Content of Jujube Fruit

In our previous study, an HPLC-ELSD method for the simultaneous determination of triterpenic acids was established [34]. After minor revision of the preparation method for the sample solution, this method was applied in this experiment to determine 10 triterpenic acids, including ceanothic acid, alphitolic acid, maslinic acid, corosolic acid, betulinic acid, oleanolic acid, ursolic acid, betulonic acid, oleanonic acid and ursonic acid. The linearity, LOD, LOQ, intra-day and inter-day precisions, stability, and accuracy were selected for the validation of the proposed method, and the results are summarized in Supplementary Materials Tables S1 and S2. A typical chromatogram of the sample and mixed standards is presented in Supplementary Materials Figure S1. The validation results revealed that the proposed method was acceptable to determine the triterpenic acids in the fruits processed with different methods.

The triterpenic acid contents of the samples (for information about the processing methods and moisture contents see Table 1) are listed in Table 2. Ursonic acid was not detected in any of the samples. On the whole, the total content of triterpenic acids showed an increasing trend during the first 24 h of the drying process, and then the content change was not significant. As for the different types of triterpenic acids analyzed in the experiment, the levels of lupane-type (alphitolic acid, betulinic acid and betulonic acid) and ursane-type compounds (corosolic acid and ursolic acid) increased significantly during the first 24 h of drying process, especially for corosolic acid and betulonic acid, which contents increased more than two-fold. 

**Table 1:** The information of the samples preparation and the moisture contents of the samples.

Sample No.	Process Method	Moisture Content (%)
S1	Fresh sample	69.5
S2	Drying at 45 °C for 24 h	48.5
S3	Drying at 45 °C for 48 h	35.7
S4	Drying at 45 °C for 72 h	28.2
S5	Drying at 45 °C for 96 h	17.1
S6	Drying at 45 °C for 120 h	14.8
S7	Drying at 45 °C for 144 h	10.2
S8	Steaming for 30 min with fresh sample	68.3
S9	Steaming for 30 min with the sample S5	33.2

**Table 2:** The analysis results of triterpenic acids in the samples (mg/100 g, DW ^1^, *n* = 3).

Sample No.	Ceanothic Acid	Alphitolic Acid	Maslinic Acid	Corosolic Acid	Betulinic Acid	Oleanolic Acid	Ursolic Acid	Betulonic Acid	Oleanonic Acid	Ursonic Acid	Total
S1 ^2^	2.71 ± 0.12 ^3,b,c,d,4^	35.82 ± 1.25 ^e^	27.47 ± 0.84 ^e^	5.81 ± 0.19 ^f^	29.06 ± 0.84 ^e^	10.88 ± 0.31 ^c,d^	nd ^5^	76.60 ± 2.99 ^f^	33.97 ± 1.29 ^c,d^	nd	222.33 ± 5.39 ^c^
S2	2.33 ± 0.05 ^f^	46.24 ± 1.51 ^a^	38.64 ± 1.02 ^a^	12.05 ± 0.38 ^b^	39.22 ± 0.97 ^c^	11.31 ± 0.40 ^b,c^	2.07 ± 0.05 ^b^	159.23 ± 5.30 ^b^	33.10 ± 1.33 ^d^	nd	344.18 ± 7.92 ^b^
S3	2.75 ± 0.08 ^b,c^	44.87 ± 1.43 ^a,b^	33.72 ± 0.99 ^d^	12.71 ± 0.26 ^a^	37.66 ± 0.59 ^c,d^	12.09 ± 0.28 ^a^	1.96 ± 0.06 ^c^	175.90 ± 4.28 ^a^	38.31 ± 1.26 ^a^	nd	359.96 ± 8.21 ^a^
S4	2.53 ± 0.07 ^e^	43.56 ± 1.65 ^b,c^	34.17 ± 1.22 ^d^	11.47 ± 0.34 ^c^	42.61 ± 1.52 ^b^	11.66 ± 0.34 ^a,b^	2.33 ± 0.06 ^a^	151.69 ± 5.18 ^c,d^	32.66 ± 0.99 ^d^	nd	332.67 ± 8.93 ^b^
S5	2.59 ± 0.09 ^d,e^	42.31 ± 1.20 ^c^	35.42 ± 0.91 ^c,d^	10.91 ± 0.31 ^d,e^	41.45 ± 0.97 ^b^	11.96 ± 0.30 ^a^	1.86 ± 0.07 ^d^	160.36 ± 4.52 ^b^	36.58 ± 0.78 ^a,b^	nd	343.43 ± 9.99 ^b^
S6	2.82 ± 0.11 ^a,b^	42.30 ± 1.41 ^c^	33.71 ± 0.89 ^d^	11.82 ± 0.28 ^b,c^	43.33 ± 1.25 ^b^	11.82 ± 0.26 ^a,b^	2.36 ± 0.06 ^a^	156.75 ± 4.01 ^b,c^	32.48 ± 1.04 ^d^	nd	337.37 ± 7.85 ^b^
S7	2.94 ± 0.09 ^a^	46.92 ± 1.29 ^a^	37.32 ± 1.30 ^a,b^	12.77 ± 0.33 ^a^	47.59 ± 1.66 ^a^	10.68 ± 0.34 ^d,e^	2.41 ± 0.05 ^a^	148.34 ± 3.18 ^d^	34.29 ± 1.28 ^c,d^	nd	343.27 ± 9.19 ^b^
S8	2.66 ± 0.06 ^c,d,e^	39.89 ± 1.05 ^d^	33.90 ± 1.01 ^d^	10.71 ± 0.28 ^e^	36.99 ± 1.33 ^d^	10.24 ± 0.27 ^e^	1.97 ± 0.07 ^c^	124.00 ± 3.59 ^e^	34.21 ± 0.96 c^,d^	nd	294.57 ± 7.87 ^b^
S9	2.62 ± 0.10 ^c,d,e^	43.54 ± 0.98 ^b,c^	36.12 ± 0.87 ^b,c^	11.45 ± 0.41 ^c,d^	42.89 ± 1.41 ^b^	11.09 ± 0.39 ^c^	2.02 ± 0.04 ^b,c^	156.67 ± 4.62 ^b,c^	35.93 ± 1.44 ^b,c^	nd	342.33 ± 8.39 ^b^

^1^ The concentrations were expressed with dry weight; ^2^ The sample no. is same as Table 1; ^3^ The data was expressed as mean ± SD; ^4^ Values in same column marked by the different letters are significantly different at *p* < 0.05; ^5^ Not detected.

Similarly, the content of total triterpenic acid in the sample steamed for 30 min (S8) was higher significantly than the fresh sample (S1). However, the dried jujube (S5) showed no significant increase of total triterpenic acid content after being steamed for 30 min. The results suggest that triterpenic acids contained in fresh jujube fruits may conjugate and could be dissociated by the enzyme**s** involved in the first 24 h of the drying process or the steaming process. With the extension of drying time, the dissociation of the triterpenic acids reaches an equilibrium and their contents no longer increased, which could also serve to explain the results that no significant increase of the triterpenic acid contents in the dried sample was noted after steaming of 30 min. Besides, the triterpenic acid content of sample S9 showed no significant difference compared to that of S5 which could also suggest that these compounds are stable to the high temperature drying treatment at 100 °C. Based on the fact that the total triterpenic acid content in the dried or steamed samples was higher than that in fresh samples, it may be a good choice for consumers to select the dried or steamed jujube fruits if triterpenic acids are their main target compounds.

### 2.2. Nucleoside and Nucleobase Contents of Jujube Fruit

Nucleosides and nucleobases were analyzed by the procedure of our previous report [35] after a minor modification of the preparation method for sample solution, which was validated and confirmed to be acceptable to determine nine nucleosides and nucleobases in the assay, including uracil, hypoxanthine, guanine, cytidine, uridine, adenine, guanosine 3′,5′-cyclic monophosphate (cGMP), guanosine and adenosine 3′,5′-cyclic monophosphate (cAMP). The results of linearity, LOD, LOQ, intra-day and inter-day precisions, stability, and accuracy are summarized in Supplementary Materials Tables S3 and S4, and a typical chromatogram of the sample and mixed standard are presented in Supplementary Materials Figure S2.

Table 3 lists the analysis results of individual nucleosides and nucleobases in jujube fruits treated by different methods. The results reveal that the total content of the nine analytes showed an increasing trend as the drying time was extended, which increased to 1051.89 μg/g in the dried fruits after drying 120 h, was more than 2-fold that in the fresh fruits (509.59 μg/g). 

As for individual compounds, the increasing content trend during the drying treatment was most obvious for the cyclic nucleotides including cAMP and cGMP, which increased more than three times (for cAMP) and four times (for cGMP), respectively. Similar varying trends were also found for other compounds except adenine and hypoxanthine. It is known that nucleosides and nucleobases in the plant body could exist as combined forms with other components, such as the formation of RNA or phosphate ester structures. With the drying treatment at 45 °C, the enzyme system existing in the fresh jujube fruit could be activated, which promoted the dissociation of these combined compounds.

Furthermore, in comparison to the fresh fruit (S1), the contents of cyclic nucleotides (cAMP and cGMP) in the steamed fruit (S8) dropped to 100.97 μg/g and 10.01 μg/g, respectively, and nearly 30% of the cAMP and 20% of the cGMP were lost during the steaming treatment. A similar trend was also found in the steaming treatment of the dried fruits, in which the content of cAMP was reduced from 423.35 μg/g (S5) to 305.42 (S9), and the content of cGMP was reduced from 45.07 μg/g (S5) to 32.68 μg/g (S9), so nearly 27% of these two cyclic nucleotides was lost. These results suggest that the cyclic nucleotides contained in the jujube fruits were not stable during steaming treatment, therefore, to avoid the loss of these healthy compounds, jujube fruits should not be steamed before consumption. Besides, considering the fact that the contents of most of nucleosides and nucleobases in the dried jujube fruits were significantly higher than those of fresh ones, it would be a good choice for consumers to select the dried ones.

**Table 3:** The analysis results of nucleosides and nucleobases in the samples (μg/g, DW ^1^, *n* = 3).

Sample No.	Uracil	Hypoxanthine	Guanine	Cytidine	Uridine	Adenine	cGMP	Guanosine	cAMP	Total
S1 ^2^	82.36 ± 1.25 ^3^^,^^e,4^	5.14 ± 0.11 ^a^	76.56 ± 1.25 ^d^	12.54 ± 0.22 ^h^	38.88 ± 0.56 ^f^	63.38 ± 1.35 ^d,e,f^	12.23 ± 0.21 ^g^	73.94 ± 1.31 ^e^	144.56 ± 1.99 ^f^	509.59 ± 7.62 ^e^
S2	88.39 ± 1.33 ^d^	4.07 ± 0.13 ^e^	50.88 ± 1.06 ^e^	14.86 ± 0.30 ^g^	39.97 ± 0.92 ^e,f^	62.86 ± 1.62 ^e,f^	15.03 ± 0.19 ^f^	79.97 ± 0.67 ^d^	192.52 ± 2.56 ^e^	548.55 ± 8.32 ^d^
S3	130.62 ± 2.69 ^c^	3.89 ± 0.06 ^e^	52.01 ± 0.68 ^e^	16.91 ± 0.21 ^f^	43.01 ± 0.65 ^d^	62.25 ± 0.56 ^f^	21.53 ± 0.32 ^e^	95.56 ± 1.11 ^c^	307.41 ± 4.62 ^d^	733.19 ± 12.30 ^c^
S4	143.04 ± 2.35 ^a,b^	4.46 ± 0.08 ^d^	111.42 ± 0.99 ^c^	18.82 ± 0.16 ^e^	53.74 ± 0.49 ^c^	69.19 ± 0.88 ^b^	39.47 ± 0.67 ^c^	96.21 ± 0.90 ^c^	363.98 ± 6.30 ^c^	900.33 ± 9.31 ^b^
S5	146.31 ±2.08 ^a^	4.76 ± 0.13 ^c^	144.38 ± 1.98 ^a^	30.65 ± 0.62 ^d^	61.32 ± 1.01 ^b^	73.09 ± 1.62 ^a^	45.07 ± 1.21 ^b^	115.98 ± 1.22 ^a^	423.35 ± 4.01 ^b^	1044.91 ± 9.68 ^a^
S6	143.49 ± 2.99 ^a,b^	4.87 ± 0.10 ^b,c^	144.43 ± 3.14 ^a^	35.34 ± 0.59 ^a^	73.37 ± 0.68 ^a^	65.78 ± 0.66 ^c^	50.99 ± 0.92 ^a^	94.73 ± 1.31 ^c^	438.89 ± 6.35 ^a^	1051.89 ± 12.37 ^a^
S7	139.49 ± 3.58 ^b^	4.85 ± 0.11 ^b,c^	141.63 ± 2.63 ^a,b^	33.74 ± 0.94 ^b^	72.96 ± 1.53 ^a^	64.89 ± 0.54 ^c,d^	51.07 ± 0.74 ^a^	94.57 ± 0.91 ^c^	439.09 ± 9.69 ^a^	1042.29 ± 9.66 ^a^
S8	84.09 ± 2.36 ^e^	5.03 ± 0.14 ^a,b^	75.42 ± 0.97 ^d^	13.12 ± 0.16 ^h^	40.74 ± 0.85 ^e^	64.22 ± 1.20 ^c,d,e^	10.01 ± 0.16 ^h^	74.58 ± 0.56 ^e^	100.97 ± 1.23 ^g^	468.18 ± 6.31 ^f^
S9	143.93 ± 1.98 ^a^	4.94 ± 0.09 ^b,c^	140.06 ± 1.35 ^b^	31.98 ± 0.87 ^c^	60.22 ± 0.74 ^b^	70.09 ± 0.99 ^b^	32.68 ± 0.65 ^d^	114.07 ± 0.98 ^b^	305.42 ± 4.91 ^d^	903.39 ± 14.02 ^b^

^1^ The concentrations were expressed with dry weight; ^2^ The sample no. is same as Table 1; ^3^ The data was expressed as mean ± SD; ^4^ Values in same column marked by the different letters are significantly different at *p* < 0.05.

### 2.3. Saccharide Content of Jujube Fruit

Saccharides were analyzed with an HPLC-ELSD method and typical chromatograms of the sample and mixed standard are presented in Supplementary Materials Figure S3. The method validation results are summarized in Supplementary Materials Tables S5 and S6, which reveal that this HPLC-ELSD method was acceptable to determine the sugars in the jujube fruits.

The determination results showed that the sugars contained in jujube fruit are mainly sucrose, glucose and fructose, which is in accordance with a previous report [32]. The contents of these sugars in the samples are listed in Table 4. Fructose and glucose were found to be the dominant sugars in all samples analyzed, while sucrose levels were very low, which is in accordance with the reference and our own previous report [32,36].

**Table 4:** The analysis results of saccharides in the samples (%, DW ^1^, *n* = 3).

Sample No.	Sucrose	Glucose	Fructose
S1 ^2^	7.92 ± 0.22 ^3,a,4^	30.42 ± 1.01 ^c^	31.15 ± 0.85 ^d^
S2	5.02 ± 0.19 ^b^	31.33 ± 0.67 ^b^	32.56 ± 1.10 ^c^
S3	3.22 ± 0.14 ^c^	32.95 ± 0.98 ^a,b^	33.74 ± 0.86 ^c^
S4	2.03 ± 0.07 ^e^	33.10 ± 1.14 ^a,b^	37.29 ± 0.92 ^a,b^
S5	nd ^5^	33.92 ± 0.87 ^a^	38.16 ± 1.21 ^a^
S6	nd	34.39 ± 0.95 ^a^	37.83 ± 1.03 ^a^
S7	nd	34.50 ± 1.23 ^a^	36.01 ± 0.88 ^b^
S8	2.56 ± 0.11 ^d^	33.25 ± 1.07 ^a^	33.58 ± 0.93 ^c^
S9	nd	34.58 ± 1.29 ^a^	37.66 ± 1.07 ^a,b^

^1^ The concentrations were expressed with dry weight; ^2^ The sample no. is the same as in Table 1; ^3^ The data was expressed as mean ± SD; ^4^ Values in same column marked by the different letters are significantly different at *p* < 0.05; ^5^ Not detected.

As shown in Table 4, a slow increasing trend was found for the content of glucose in the jujube fruits during the drying treatment, after which its content increased nearly 15%. Fructose content gradually increased during the drying treatment, and reached a maximum after drying for 96 h, then it decreased as the drying time was extended further. Sucrose exhibited a decreasing trend and its content was reduced from 7.92 μg/g in the fresh sample (S1) to being not detectable in the samples after drying for 96 h (S5–S7). As for the fresh jujube fruits, their sucrose content showed a significant decrease after being steamed for 30 min, and nearly 68% of the sucrose was lost in this treatment. Interestingly, an opposite trend was found for the levels of fructose and glucose, which all showed a slight increase in the steamed sample compared to the fresh jujube fruit, while no statistically significant difference were found for the dried samples after being steamed. It is known that sucrose can be hydrolyzed to produce fructose and glucose by enzymatic catalysis or under acid conditions, which could explain the result of this assay that the content of sucrose was decreasing, while the contents of fructose and glucose was increasing during the drying treatment. Besides, fructose has a higher relative sweetness than glucose. Thus, the perception of sweetness in jujube fruits is likely due to the prevalence of fructose, especially for the dried fruits due to the absence of sucrose.

## 3. Experimental Section

### 3.1. Chemicals

The standards of triterpenic acids including ceanothic acid, alphitolic acid, maslinic acid, corosolic acid, betulinic acid, oleanolic acid, ursolic acid, betulonic acid, oleanonic acid, and ursonic acid were isolated from *Z. jujuba* fruits in our laboratory, and their structures were identified by NMR, HPLC and MS. Reference compounds such as sucrose, d-glucose, d-fructose, uracil, hypoxanthine, guanine, uridine, adenine and cAMP were obtained from the National Institute for the Control of Pharmaceutical and Biological Products (Beijing, China). Cytidine, cGMP, and guanosine were purchased from Sigma (St. Louis, MO, USA). Figure 1 presents the structures of all the reference compounds mentioned above. The purities of all these analytes are more than 98% as determined by HPLC. The methanol, acetonitrile, and ammonium acetate used were of HPLC grade and obtained from Merck (Darmstadt, Germany). Deionized water was prepared by a water superpurification system (Eped, Nanjing, China). Other reagent solutions were all of analytical grade and purchased from Sinopharm Chemical Reagent Co., Ltd. (Shanghai, China).

### 3.2. Sampling of Jujube Fruits

A local cultivar of *Z. jujuba* Mill. which is called “Lingwuchangzao” and is cultivated in Yinchuan (China) was used in this research. All trees were managed in accordance with integrated cultivation protocols, and the fruits were collected on 25 September 2013. Physiologically mature jujube fruits were picked, as defined by experienced horticulturists, based on color, flavor, and structure of the fruits. A total weight of 10 kg of jujube fruits from 20 jujube trees were collected and separated into three batches (F1, F2 and F3). The sample S1 was obtained from the fresh fruits of F1. The fresh sample F2 was subjected to a drying process at 45 °C in a hot air circulation oven, and sampled every 24 h until the moisture content reach 10%, at which point the samples S2 to S7 were obtained; F3 was steamed for 30 min in a closed pot to produce sample S8; The sample S5 obtained with the hot air circulation oven drying method with a moisture content between 15%–20% was selected for steaming for 30 min in a closed pot, and thereby sample S9 was obtained. Each experiment was carried out in triplicate. All the samples were broken into granules with less than 3 mm in diameter, and the moisture contents determined by the method of drying in an oven at 105 °C. The sample processing information is summarized in Table 1.

### 3.3. HPLC-ELSD Analysis of Triterpenic Acids

Triterpenic acids were analyzed with a modified procedure from our previous study [34]. A total of 20–40 jujube fruits with uniform size from each drying stage were selected for analysis. The fruits were divided into pulps and shells with a knife. The pulps were then cut with a knife into thin strips (2 mm × 2 mm) and homogenized with a homogenizer. A portion of the jujube pulp (approximately 2.5 g of dry weight) was selected and weighed accurately into a 100 mL conical flask equipped with a stopper and then extracted two times (30 min each) with ultrasound assistance using 50 mL of chloroform. The extracts were combined and filtered through analytical filter paper, then evaporated to dryness with a rotary evaporator. The residue was dissolved with methanol in a 10 mL volumetric flask, and then filtered through a 0.45 μm membrane filter before injection into the HPLC system for analysis.

Samples were analyzed on a Waters 2695 Alliance HPLC system (Waters Corp., Milford, MA, USA), which includes a quaternary pump solvent management system, an online degasser, and an autosampler. A Waters 2424 ELSD was used to detect the raw data which was acquired and processed with Empower software. A Hypersil C18 column (250 mm × 4.6 mm, 5 μm) preceded by a Waters Symmetry Shield RP C18 guard column (20 mm × 3.9 mm, 5 μm) was used for all analyses. The mobile phase was composed of A (MeOH) and B (0.3% acetic acid and 0.15% triethylamine in H_2_O, *v*/*v*) with a gradient elution: 0–15 min, 75%–87% A; 15–45 min, 87% A; 45–50 min, 87%–100% A. Re-equilibration duration was 15 min between individual runs. The injection volume was set as 10 μL except for the determination of ursolic acid which was set as 20 μL. The flow rate of the mobile phase was set as 0.5 mL/min, and the column temperature was maintained at 25 °C. The drift tube temperature of the ELSD was set at 80 °C, and the nitrogen flow rate was 2.7 L/min. Quantification was performed according to the linear calibration plots of the logarithm of peak areas *vs.* the logarithm of concentration. The concentrations were expressed in milligrams per 100 g of dry weight (DW).

### 3.4. UHPLC Analysis of Nucleosides and Nucleobases

A modified procedure from our previous research [35] was used to analyze the nucleosides and nucleobases. A portion of the jujube pulp (approximately 2.0 g by dry weight) was selected and extracted with 40 mL of water for 30 min in an ultrasonic bath (40 kHz) at room temperature. Then the same solvent was added to compensate for any weight lost during the extraction. After centrifugation (13,000 rpm, 10 min), the supernatant was stored at 4 °C and filtered through a 0.22 μm membrane filter before injection into the UHPLC system for analysis.

Samples were analyzed on a Waters Acquity UPLC system, which includes a quaternary pump solvent management system, an autosampler, an online degasser, and an Acquity photodiode array detector. The separation was performed on an Acquity UPLCHSS T3 (100 mm × 2.1 mm, 1.8 μm) column. MassLynx 4.1 software (Waters Corp.) was used to acquire and process the raw data. The mobile phase was composed of A (MeOH) and B (5 mM ammonium acetate solution, adjusted to pH 8.0 with ammonia–water) with a gradient elution: 0–3 min, 0% A; 3–7 min, 0%–6% A; 7–10 min, 6%–15% A, 10–11 min, 15%–50% A. The flow rate of the mobile phase was 0.3 mL/min, and the column temperature was maintained at 30 °C. Detection wavelength was set at 273 nm for guanine, 269 nm for cytidine, and 254 nm for the others. The quantification was performed with integrated chromatographic peak areas from each jujube sample comparing with the peak areas of known amounts of standard sample. The results of concentrations were displayed with micrograms per gram of DW.

### 3.5. HPLC-ELSD Analysis of Sugars

The contents of saccharides including sucrose, glucose, and fructose in the samples were analyzed in this assay with a modified version of a reported LC method [36,37]. A portion of the jujube pulp (approximately 1.0 g of dry weight) was selected and extracted with 100 mL of water in an ultrasonic bath (40 kHz) at room temperature for 60 min. Then the extract was centrifugalized (15,000× *g*, 10 min) and the supernatant was separated and transferred to vials.

Analysis was performed on a Waters 2695 Alliance HPLC system and a Carbo Sep CHO-682 Pb column (7.8 × 300 mm, 7 μm) (Transgenomic, Inc., San Jose, CA, USA) with deionized water as the mobile phase was used to separate the saccharides. The flow rate and the column temperature were optimized as 0.4 mL/min and 65 °C, respectively. The injection volume was 10 μL. The detection was performed on a Waters 2424 ELSD with the drift tube temperature of 80 °C and nitrogen flow-rate of 2.7 L/min, respectively. The quantification was performed according to linear calibration plots of the logarithm of peak areas *versus* the logarithm of concentrations. The concentrations were displayed with grams per 100 g of DW.

### 3.6. Statistical Analysis

In this study, all experiments were carried out at least three times. The analysis results were presented as mean ± SD. One-way ANOVA analysis was used to statistically evaluate the data with the aid of SPSS 18.0 software (SPSS Inc., Chicago, IL, USA). When significant difference presented, the least significant difference (LSD) test was utilized to analyze the differences among means. All of the statistical differences were carried out at a significance level of α = 0.05.

## 4. Conclusions

To our knowledge, this is the first report to investigate the variations of the contents of triterpenic acids, nucleosides and nucleobases, and saccharides in jujube fruits during the drying and steaming treatment. The results showed that except for sucrose, the content levels of most analytes determined in the study were increased when the jujube fruits were dried at 45 °C. Therefore, considering the bioactivities and beneficial effects of these components for human health, the dried fruits would be the better choice as medicinal materials or functional foods. Considering the fact that the level of cyclic nucleotides was significantly decreased after the dried fruits were steamed, and no statistically significant difference were found for other components in this process, dried jujube fruit should not be further steamed. Additionally, although change trends of these components were observed during the drying and steaming treatment, their variation mechanisms are not clear and need to be researched in future studies.

## Supplementary Materials

Supplementary materials can be accessed at: http://www.mdpi.com/1420-3049/20/12/19852/s1.
