# Supplementary material for: Contents Changes of Triterpenic Acids, Nucleosides, Nucleobases, and Saccharides in Jujube (Ziziphus jujuba) Fruit During the Drying and Steaming Process
Source: Molecules. 2015 Dec 12;20(12):22329–40. doi: 10.3390/molecules201219852 (PMC6332246; doi:10.3390/molecules201219852)
Supplement: Supplementary file 1 [file molecules-20-19852-s001.pdf]

molecules

MDPI

Supplementary Materials: Contents Changes of Triterpenic Acids, Nucleosides, Nucleobases, and Saccharides in Jujube (*Ziziphus jujuba*) Fruit During the Drying and Steaming Process

Sheng Guo, Jin-ao Duan, Ying Zhang, Dawei Qian, Yuping Tang, Zhenhua Zhu and Hanqing Wang

Table S1. Calibration curves, LOD and LOQ data of the investigated triterpenic acids by HPLC-ELSD.

Analyte	Calibration Curves [a]	r^2	Linear Range (μg/mL)	LOD [b] (μg/mL)	LOQ (μg/mL)
Ceanothic acid	y = 1.6327x + 2.2567	0.9987	3.890–311.2	1.94	3.89
Alphitolic acid	y = 1.6120x + 2.2142	0.9986	12.24–979.2	2.45	4.90
Maslinic acid	y = 1.6025x + 2.1014	0.9990	11.98–958.4	3.20	5.99
Corosolic acid	y = 1.6535x + 2.2431	0.9995	7.680–614.4	3.84	7.68
Betulinic acid	y = 1.6538x + 1.8978	0.9989	12.15–972.0	3.43	6.85
Oleanolic acid	y = 1.6196x + 2.0233	0.9987	11.58–926.4	3.86	7.72
Ursolic acid	y = 1.5901x + 2.4257	0.9992	10.02–801.6	3.34	6.68
Betulonic acid	y = 1.6434x + 1.8524	0.9995	13.02–1042	4.34	8.68
Oleanonic acid	y = 1.6425x + 1.9150	0.9993	14.98–1198	4.99	9.98
Ursonic acid	y = 1.6489x + 1.9481	0.9990	14.05–1124	4.68	9.36

[a] Y is the logarithmic value of peak area, and X is the logarithmic value of the reference compound's concentration (μg/mL); [b] LOD and LOQ were determined at the S/N of about 3 and 10, respectively.

Table S2. Precision, repeatability, stability, and recovery of the investigated triterpenic acids by HPLC-ELSD.

Analyte	Precision (RSD, %)		Repeatability (RSD, %, n = 6)	Stability (RSD, %, n = 6)	Recovery (%, n= 3)	
	Intra-Day (n = 6)	Inter-Day (n = 6)			Mean	RSD
Ceanothic acid	2.0	2.6	3.2	2.8	97.5	2.0
Alphitolic acid	1.9	2.1	2.5	2.6	96.8	2.9
Maslinic acid	2.2	2.7	2.7	2.2	98.0	3.1
Corosolic acid	2.5	2.9	3.3	3.0	102.4	3.3
Betulinic acid	1.7	2.8	2.8	2.7	96.9	3.1
Oleanolic acid	2.1	3.1	2.9	2.2	96.4	2.7
Ursolic acid	1.9	1.9	2.4	2.9	100.3	2.9
Betulonic acid	2.6	2.7	2.7	3.0	96.2	2.4
Oleanonic acid	2.3	2.4	3.0	3.2	95.1	3.2
Ursonic acid	2.8	3.0	-	-	-	-

Molecules **2015**, *20*, S1–S4; doi:10.3390/molecules201219852

Table S3. Calibration Curves, LOD and LOQ Data of the Investigated nucleobases and nucleosides by UPLC-DAD.

Analyte	Calibration Curves [a]	r^2	Linear Range (μg/mL)	LOD [b] (μg/mL)	LOQ (μg/mL)
Uracil	y = 432.82x + 10.15	0.9997	0.12-12.05	0.024	0.070
Hypoxanthine	y = 1056.32x − 10.54	0.9999	0.11-10.95	0.011	0.040
Guanine	y = 158.67x + 19.42	0.9998	0.15-15.12	0.030	0.098
Cytidine	y = 432.16x + 7.57	0.9995	0.19-19.13	0.038	0.120
Uridine	y = 457.32x − 54.07	0.9996	0.49-49.32	0.025	0.060
Adenine	y = 1143.63x − 24.52	0.9997	0.15-14.95	0.015	0.050
cGMP	y = 458.65x + 7.94	0.9999	0.10-10.45	0.021	0.063
Guanosine	y = 606.85x − 7.85	0.9998	0.20-20.10	0.020	0.070
cAMP	y = 637.68x + 22.33	0.9999	0.50-49.58	0.015	0.045

[a] Y is the value of peak area, and X is the value of the reference compound's concentration (μg/mL);
[b] LOD and LOQ were determined at the S/N of about 3 and 10, respectively.

Table S4. Precision, repeatability, stability and recovery of nucleobases and nucleosides by UPLC-DAD.

Analyte	Precision (RSD, %)		Repeatability (RSD, %, *n* = 6)	Stability (RSD, %, *n* = 6)	Recovery (%, *n* = 3)	
	Intra-Day (*n* = 6)	Inter-Day (*n* = 6)			Mean	RSD
Uracil	0.84	1.68	1.58	1.32	98.1	1.98
Hypoxanthine	0.43	0.85	2.01	1.54	102.3	2.05
Guanine	0.52	1.56	1.67	1.89	96.6	2.22
Cytidine	0.31	1.32	1.59	0.98	98.3	1.68
Uridine	0.33	2.05	1.32	0.65	99.6	1.76
Adenine	0.40	1.64	2.09	0.79	97.5	1.88
cGMP	0.39	2.02	0.98	2.15	101.3	2.12
Guanosine	0.56	1.55	0.65	1.21	97.2	2.06
cAMP	0.22	0.94	1.87	2.24	99.4	2.05

Table S5. Linear regression data, LOD and LOQ of the investigated sugars by HPLC-ELSD.

Analyte	Calibration Curves [a]	r^2	Linear Range (μg/mL)	LOD [b] (μg/mL)	LOQ (μg/mL)
Sucrose	y = 1.5811x + 1.6259	0.9982	108.2-2164	54.1	108.2
Glucose	y = 1.5845x + 1.8763	0.9991	248.3-4966	30.1	60.2
Fructose	y = 1.5789x +2.1956	0.9979	241.2-4824	40.2	80.4

[a] y is the logarithmic value of peak area and x is the logarithmic value of the reference compound's concentration (μg/mL); [b] LOD and LOQ were determined at the S/N of about 3 and 10, respectively.

Table S6. Precision, repeatability, stability and recovery of the investigated sugars by HPLC-ELSD.

Analyte	Precision (RSD, %)		Repeatability (RSD, %, *n* = 6)	Stability (RSD, %, *n* = 6)	Recovery (%, *n* = 3)	
	Intra-Day (*n* = 6)	Inter-Day (*n* = 3)			Mean	RSD
Sucrose	2.04	4.36	2.30	3.69	97.38	2.87
Glucose	2.31	3.11	1.95	1.99	102.10	3.26
Fructose	2.54	3.20	2.07	2.70	96.35	3.99

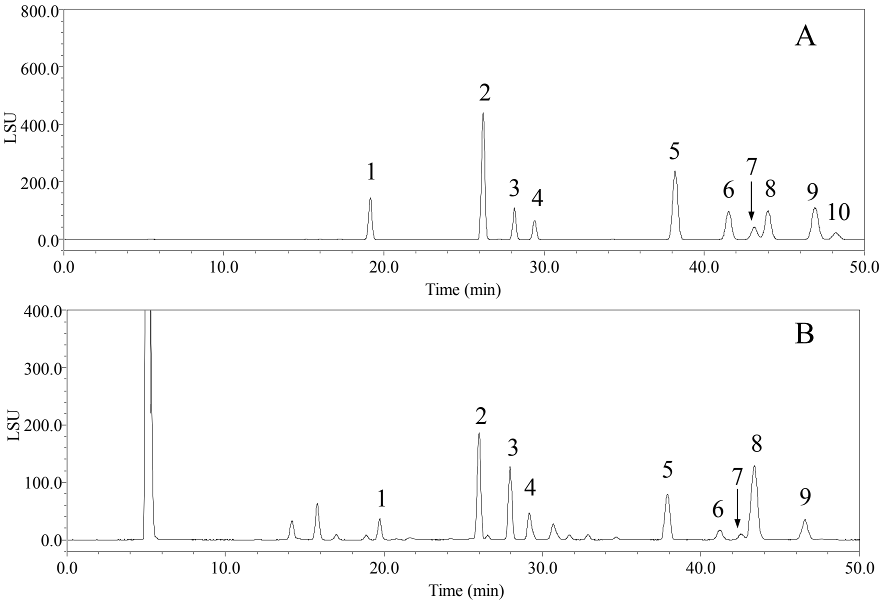

Figure S1. Typical HPLC-ELSD chromatograms of triterpenic acids in mixed standards (**A**) and sample of jujube fruits (**B**). Peak identities: ceanothic acid (**1**), alphitolic acid (**2**), maslinic acid (**3**), corosolic acid (**4**), betulinic acid (**5**), oleanolic acid (**6**), ursolic acid (**7**), betulonic acid (**8**), oleanonic acid (**9**), ursonic acid (**10**).

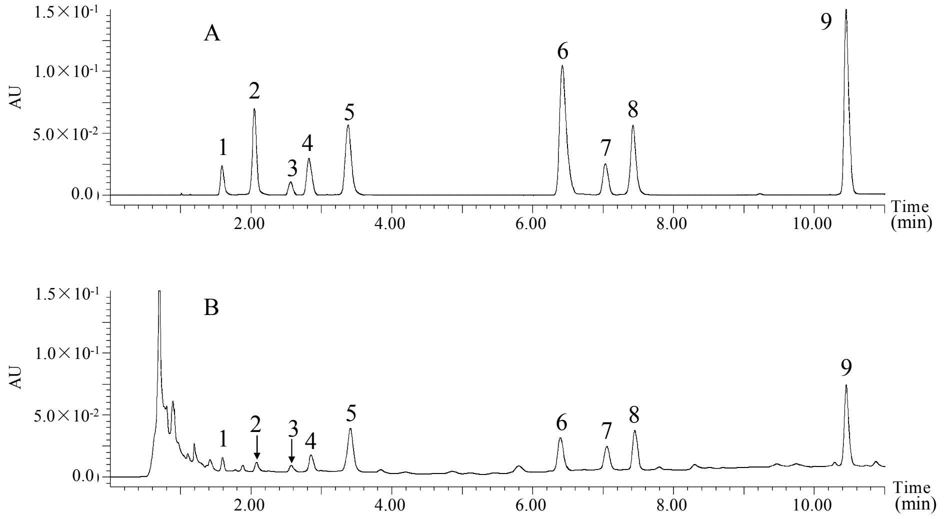

Figure S2. Typical UHPLC-DAD chromatogramsof nucleosides and nucleobases in mixed standards (A) and sample of jujube fruits (B). Peak identities: uracil (**1**), hypoxanthine (**2**), guanine (**3**), cytidine (**4**), uridine (**5**), adenine (**6**), cGMP (**7**), guanosine (**8**), cAMP (**9**).

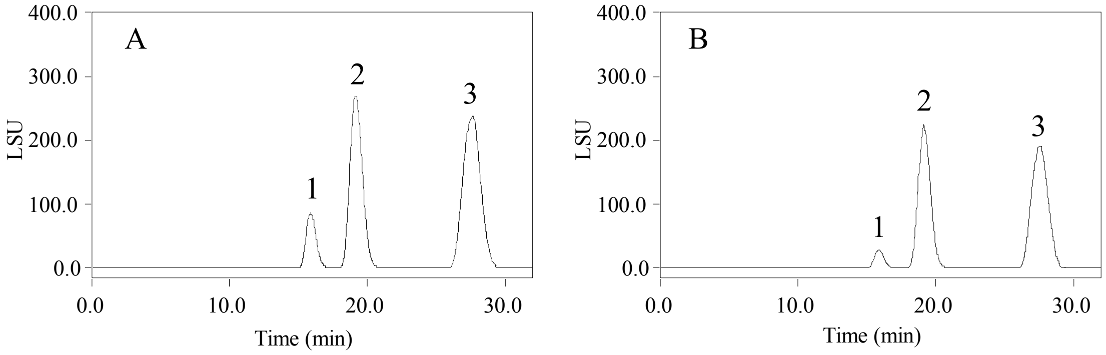

Figure S3. Typical HPLC-ELSD chromatograms of sugars in mixed standards (**A**) and sample solutions (**B**). Peak identities: sucrose (**1**), glucose (**2**), fructose (**3**).
